# The limited clinical utility of a routine creatine kinase (CK) on admission to a psychiatric inpatient unit

**DOI:** 10.1186/s12888-024-06386-8

**Published:** 2024-12-18

**Authors:** Fraser A. M. Scott, Matt Butler, Jonathan P. Rogers

**Affiliations:** 1 South London and Maudsley NHS Foundation Trust, London, UK; 2 Institute of Psychiatry, Psychology & Neuroscience, King’s College London, London, UK; 3 Division of Psychiatry, University College London, London, UK; 4 Mental Health Liaison Service, St Thomas’ Hospital, Westminster Bridge Road, London, SE1 7EH UK

**Keywords:** Neuroleptic malignant syndrome, NMS, Creatine kinase, CK, Screening

## Abstract

**Background:**

Creatine kinase (CK) is an intracellular enzyme expressed most commonly in tissues such as skeletal muscle. CK can be used as an investigation to support the diagnosis of conditions such as neuroleptic malignant syndrome (NMS), a rare idiosyncratic drug reaction – classically to antipsychotic medications – which can be fatal. Routine screening of CK in psychiatric inpatients is a known practice, but its value is uncertain. We aimed to ascertain whether such screening resulted in new diagnoses of NMS or other conditions, and changes in clinical management.

**Methods:**

Using an electronic case register, we conducted a descriptive retrospective cohort study, identifying all psychiatric inpatient admissions in a South London mental health trust over a four-year period where a CK test was conducted within 48 h of admission. We extracted the demographic and clinical characteristics (e.g., diagnosis) of those who met inclusion criteria. Free-text review was performed on all those with a CK potentially suggestive of NMS (CK ≥ 4x upper limit of normal reference range (ULN)) to determine the impact of this abnormal result on subsequent management and diagnosis (including NMS if identified).

**Results:**

Of 14,236 inpatient episodes in the specified window, 2358 (16.6%) had a CK test within 48 h of admission. This was ≥ 4x ULN in 327 (13.8%) cases (free-text successfully reviewed in 318). There were no cases of NMS identified. An abnormal CK result led to a new alternative diagnosis, such as dehydration or catatonia, in only 14 patients (4.4% raised CK sample, 0.6% total CK sample). Impact on subsequent management appeared limited, with the most common adjustment being an increase in frequency of physical observations in 47 instances (14.8%).

**Conclusions:**

The clinical utility of untargeted screening using a serum CK for psychiatric inpatients appears limited, with poor specificity in detection of NMS and a minimal impact on subsequent clinical management.

## Background

Creatine kinase (CK) is an intracellular enzyme chiefly located in skeletal muscle, but also present in cardiac muscle and certain brain tissues [1]. CK is released into the bloodstream when there is muscle injury, and – due to its ubiquitous nature – many conditions can therefore result in elevations in serum CK, including heart disease, renal impairment, hypoxia, or use of certain medications (e.g., statins) [2, 3]. Physiological factors can also impact CK levels, including sex, age, race, muscle mass and recent physical activity (including psychomotor agitation and restraint) [2]. Given the myriad physiological and pathological factors that can impact CK activity, it is reasonable to infer that it would have suboptimal value as a screening investigation, lacking requisite specificity.

Neuroleptic malignant syndrome (NMS) is an idiosyncratic but potentially fatal reaction to treatment with dopamine D_2_ receptor antagonists and partial agonists, most notably antipsychotic medications [4]. It is a rare complication, with data from 1993 to 2015 suggesting an incidence of 0.016% patients prescribed antipsychotics during this period across psychiatric inpatient settings [5]. The presentation of NMS is heterogeneous, both in terms of severity and symptoms expressed [6], and it has been conceived as a form of drug-induced catatonia [7]. 

The classic features of NMS are considered to be: hyperpyrexia, muscle rigidity, alterations in mental state, and autonomic instability [4, 8]. However, symptoms can often be mild, and there is considerable overlap between the signs and symptoms of NMS and the common adverse effects of antipsychotic medication (e.g., the muscle rigidity of NMS can be indistinguishable from drug-induced parkinsonism) [4]. 

Further complicating the definitive diagnosis of NMS is the lack of a biomarker with high specificity [4], which would in turn confer a greater positive predictive value, given the low disease prevalence [9]. There is some debate about the minimum threshold value of CK that is considered to be indicative of NMS [10, 11]. Moreover, elevation of CK is often a non-specific finding; abnormally elevated CK has been observed in up to 70% of inpatients with psychosis, without other features suggestive of NMS [12]. 

Routine testing of CK among psychiatric patients has been advocated by some, although there is a lack of agreement [13]. Routine CK testing is advised on admission to psychiatric units in some local health trust policies to aid in identification of NMS [14]. Nevertheless, as previously argued by the authors, indiscriminate screening with investigations lacking sufficient sensitivity and specificity can be problematic due to low yield and the potential for generation of needless anxiety [15]. Given the poor specificity and sensitivity of elevated CK as indicative of NMS, we hypothesised that routine testing on admission to psychiatric inpatient units might have limited clinical utility.

We aimed to examine the clinical utility of a screening CK investigation by identifying all inpatient episodes for a London mental health trust where a routine CK test was performed on admission, and determining if detection of an abnormally elevated CK resulted in: a new diagnosis of NMS, identification of another new physical health condition, or identifiable change in clinical management in response to an abnormal CK result. We additionally aimed to describe the clinical and laboratory features present in any suspected/confirmed cases of NMS.

## Methods

### Setting

In this study, we used the Clinical Records Interactive Search (CRIS) database. This is an anonymized database of mental healthcare records from South London and Maudsley (SLAM) NHS Foundation Trust, London, United Kingdom. It includes both inpatient and community care records for over 400,000 patients from 2006 onwards [16]. Its de-identification extends to removal of possible identifiers from free-text entries, allowing these to be manually reviewed. The CRIS database is approved by the Oxfordshire C Research Ethics Committee (ref: 23/SC/0257). This individual study was approved by the CRIS Oversight Committee (ref: 21–070).

### Study design

We conducted a retrospective descriptive cohort study, in which we identified all inpatient admissions in SLAM beginning between 1st January 2017 and 31st December 2020, inclusive. Ongoing admissions were included, as were multiple admissions of the same patient during the specified window. For each inpatient episode, we ascertained whether there had been a CK blood test conducted within 48 h of admission commencing. This was termed an “admission CK”. If this had been performed, then the individual episode met inclusion criteria. There were no exclusion criteria.

### Participants

For each inpatient episode that met the above criteria, the following demographic and clinical data were extracted as structured fields: age at admission start, date of birth, ethnicity, sex, duration of admission, diagnosis (using International Statistical Classification of Diseases and Related Health Problems 10th Revision (ICD-10) diagnostic categories) [17], and date of diagnosis. The timings and results (units/liter; U/L) of all CK tests conducted were also extracted.

### Exposure

The lowest threshold value of CK that is considered to indicate possible NMS is not conclusively established. However, an international consensus using the Delphi method considered a CK of at least 4 times the upper limit of normal (ULN) the minimum suggestive value [10]. ULN of CK was defined in our laboratory as 150U/L. Manual review of all admissions with an initial CK ≥ 4x ULN (≥ 600U/L) was therefore conducted.

In episodes with an admission CK ≥ 4x ULN (the ‘raised CK’ group), one author (F.S.) manually reviewed the de-identified clinical records to ascertain the presence and specifics of substance misuse (including positive urine drug screen (UDS)) and antipsychotic prescription on admission. Furthermore, free-text entries were reviewed to assess for any documentation of clinical features or concerns that preceded testing CK, and that would suggest that assessment of CK was clinically justifiable.

### Outcomes

In the raised CK group, we examined if the abnormal result led to: a change in diagnosis (1), a change in management (2), or a change in medication (3).

1. A change in diagnosis included: a new diagnosis (not exclusively NMS) being made, or a suspected diagnosis being confirmed.
2. Change in management included: any change in the level of physical health observations, case discussion with a colleague in another medical specialty, patient transfer to an emergency department, and/or admission to a general hospital.
3. Changes in medication included: a new medication being started, cessation/dose-adjustment of a current medication, and/or any alterations in planned treatment timeline (e.g., a new prescription of an antipsychotic being delayed).

Where cases of NMS or suspected NMS were discovered, we reviewed entries to identify documented symptoms, physical observations, and laboratory investigations. These were compared to the diagnostic criteria for NMS in the Diagnostic and Statistical Manual of Mental Disorders Fifth Edition (DSM-5) [8]. 

### Analysis

Demographic variables were presented for the whole cohort, those episodes with an initial CK < 4x ULN and those with ≥ 4X ULN using simple descriptive statistics (*n*, percentage (%)). For continuous variables, mean and standard deviation were presented when data were normally distributed and median and interquartile range when not normally distributed. A Q-Q plot was created to assess normal distribution of CK values.

## Results

### Participants

**Fig. 1:**
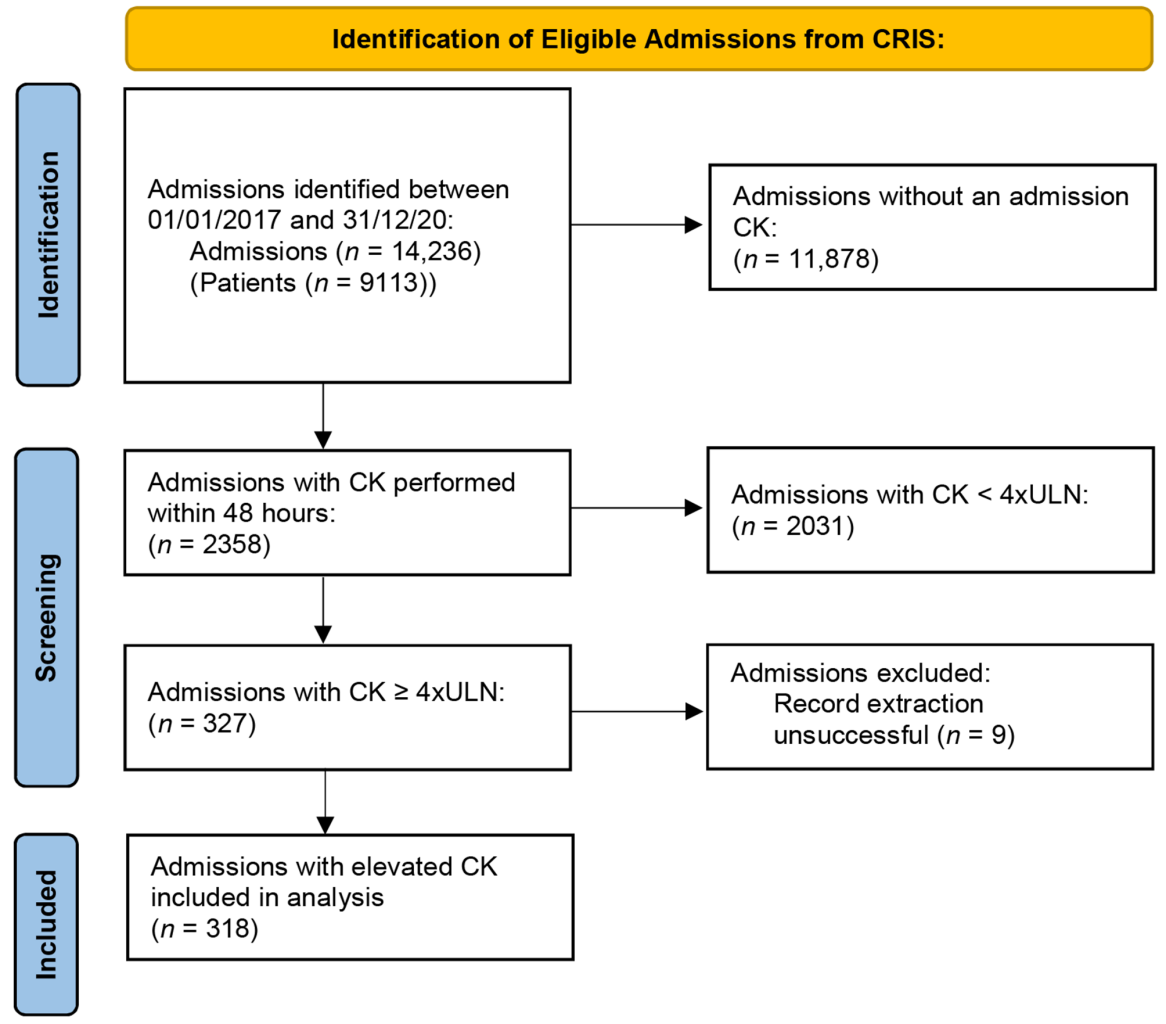
PRISMA flow diagram demonstrating process from identification to analysis for all admissions between 1st January 2017 and 31st December 2020

We identified 14,236 admissions (of 9113 individuals) during the study period of which 2358 (16.6%) had a CK performed within 48 h of admission. Of those admissions with a baseline CK, 327 (13.9%) had an initial CK ≥ 600U/L (4 x ULN) (‘raised CK’ cohort). The full process from identification to analysis is outlined in Fig. 1. There were 9 cases in which extraction of free-text information was not possible due to failure of electronic record retrieval; the information from these individuals is included in the initial demographic description of the cohort with admission CK ≥ 600U/L (*n* = 327), but not in the analysis of outcomes (*n* = 318).

### Characteristics

The characteristics of all identified admissions in addition to those with a raised CK and those with a CK < 600U/L are summarized in Table 1. The cohort with an admission CK ≥ 600U/L were on average younger (38.0 years (SD 13.7)) than those with an initial CK < 600U/L (41.1 years (SD 16)), and a higher proportion of the group were men (69.4% vs. 46.1%). As outlined in Table 1, the other characteristics of the two cohorts were similar.

**Table 1:** Full demographic characteristics of the entire cohort, and those with admission CK < 600U/L (< 4 x ULN) and those with CK ≥ 600U/L (≥ 4 x ULN). Diagnoses have been grouped into larger categories

	Whole Cohort	CK < 600U/L	Raised CK (≥ 600U/L)
**Number of admissions**	2358	2031	327
**Age (SD)**	40.7 (15.7)	41.1 (16.0)	38.0 (13.7)
**Sex**, ***n*(%)**:			
**Male**	1164 (49.4%)	937 (46.1%)	227 (69.4%)
**Female**	1194 (50.6%)	1094 (53.9%)	100 (30.6%)
**Diagnosis category**, ***n*(%)**:			
**Psychosis**	1028 (43.6%)	841 (41.4%)	187 (57.2%)
**Affective**	481 (20.4%)	404 (19.9%)	77 (23.5%)
**Substance-related**	128 (5.4%)	109 (5.4%)	19 (5.8%)
**Anxiety disorder**	130 (5.5%)	126 (6.2%)	4 (1.2%)
**Personality disorder**	268 (11.4%)	253 (12.5%)	15 (4.6%)
**Dementia**	40 (1.7%)	39 (1.9%)	1 (0.3%)
**Eating disorder**	80 (3.4%)	78 (3.8%)	2 (0.6%)
**Puerperal disorder**	14 (0.6%)	13 (0.6%)	1 (0.3%)
**Learning disability**	6 (0.3%)	5 (0.2%)	1 (0.3%)
**Neurodevelopmental disorder**	20 (0.8%)	18 (0.9%)	2 (0.6%)
**Organic (including delirium)**	32 (1.4%)	29 (1.4%)	3 (0.9%)
**Other (including unspecified and malingering)**	131 (5.6%)	116 (5.7%)	15 (4.6%)
**Duration of admission (days) (SD)**	49.8 (76.6)*	49.9 (79.4)**	48.7 (56.2)***
**Time from admission to initial CK**,** hours (SD)**	9.07 (17.23)	8.95 (17.08)	9.85 (18.17)
**Deaths during admission**, ***n*(%)**	5 (0.21%)	3 (0.15%)	2 (0.61%)

*Excluding 20 admissions that were ongoing at time of data extraction

**Excluding 19 ongoing admissions

***Excluding 1 ongoing admission

As there were 165 unique diagnoses recorded, they have been grouped in Table 1. Paranoid schizophrenia was the most common individual diagnosis in both cohorts (data not shown). As illustrated in Table 1, there was a greater proportion of psychotic and affective disorders in the raised CK cohort, but almost all other diagnoses were more prevalent in those with CK < 600U/L.

### Interventions

The outcomes for the cohort (*n* = 318) with raised admission CK (≥ 600U/L) are given in Table 2. Q-Q plot was strongly suggestive of a positively skewed distribution of CK values. The median value of the admission CK in this group was 1242U/L (IQR 1262); a repeat test was performed on 70 (22.0%) occasions, with the median value of the subsequent CK reducing to 864U/L (IQR 2527).

**Table 2:** Outlines the baseline clinical features and outcomes after a CK ≥ 600U/L (CK ≥ 4x ULN) result was returned. CK results for each inpatient episode were extracted automatically, whereas all other features/outcomes were manually compiled from free-text review

	Raised CK (≥ 600U/L)
**Number of admissions**	318
**Value of initial CK**,** median (IQR)**	1242U/L (1262)
**Number of repeated CK tests (%)**	70 (22.0%)
**Value of second CK**,** median (IQR)**	864U/L (2527)
**Baseline clinical features**:	
**Substance misuse**, ***n*(%)**	147 (46.2%)
**Antipsychotic prescription**, ***n*(%)**	119 (37.4%)
**Clinical features to justify testing CK**	43 (13.5%)
**Outcomes**:	
**New diagnosis made**, ***n*(%)**	14 (4.4%)
**Cases of NMS diagnosed**, ***n*(%)**	0 (0.0%)
**Frequency of observations increased**, ***n*(%)**	47 (14.8%)
**Medication altered/discontinued**, ***n*(%)**	17 (5.3%)
**Discussion with general medical colleague**, ***n*(%)**	43 (13.5%)
**Transfer to emergency department**, ***n*(%)**	19 (6.0%)
**Admitted to general hospital**	11 (3.5%)

IQR: interquartile range

Documentation of a clinical rationale for performing a CK test was only present on 43 (13.5%) occasions. These were varied, but the most common were: rechecking a previously raised CK (12; 3.8%), physical signs or symptoms, including pain (10; 3.1%) and abnormal physical observations (9; 2.8%). Antipsychotics were prescribed (with no noted suspicion of non-adherence) in 119 (37.4%) instances.

At point of admission, concerns about substance misuse or a positive UDS were recorded in 147 (46.2%) of inpatient episodes. Cannabis was the most commonly observed recreational substance, with use present in 94 episodes (29.6% of entire raised CK cohort), comprising 63.9% of all substance use. Polysubstance misuse was the second-most frequent observation, occurring in 35 cases (11.0% of entire raised CK cohort; 23.8% of all substance misuse).

There we no cases of NMS detected in our cohort. In 14 (4.4%) of episodes with an admission CK ≥ 600U/L (≥ 4x ULN) there were other confirmed/strongly suspected diagnoses made in the light of the result: the most common diagnoses were catatonia and infection in 3 (0.9%) patients each. There were a range of infections diagnosed with 1 (0.3%) case each of cellulitis, a urinary tract infection (UTI), and a perianal abscess. Dehydration was the only other diagnosis recorded more than once (2 (0.6%) cases). All recorded diagnoses are provided in Table 3. Of the 43 admissions with a clinical rationale for performing a CK test, a new diagnosis was made in 7 (16.3%). On identification of a CK ≥ 600U/L there was a detectable change in management in only a minority of cases (Table 2).

Of the 19 (6.0%) patients with raised admission CK who were transferred to an emergency department, 11 (57.9%) were admitted to a general hospital. 15 (78.9%) had other physical symptoms/signs in addition to an elevated CK (including abnormal observations) and 10 (52.6%) had other abnormal laboratory investigations. Other abnormal laboratory investigations recorded in multiple patients included deranged liver function tests (LFTs) in 5 (26.3%), acute kidney injury (AKI) in 3 (15.8%) and raised C-reactive protein (CRP) in 2 (10.5%).

In only 1 case was an isolated raised CK the reason for emergency department transfer. Only 6 (31.6%) of the above 19 were prescribed an antipsychotic at the time of this elevated CK, and in 5 cases (26.3%) the CK result was not commented on as part of the rationale for transfer to the emergency department.

There was a change in medication in 17 (5.3%) patients subsequent to a raised CK. This included 6 (1.9%) instances of an antipsychotic being held/discontinued. Catatonic features were present in 2 (0.6%) of these cases. In 1 (0.3%) case, the antipsychotic was held due to a decrease in renal function, rather than elevated CK. In 3 (0.9%) patients, antibiotics were commenced for a suspected infection, and on 2 (0.6%) occasions intravenous fluids were administered. No other medication alteration occurred in more than one instance.

**Table 3:** All diagnoses recorded in those (*n* = 318) with a raised initial CK (CK ≥ 4x ULN)

Diagnosis	*n*	Percentage (%)
Catatonia	3	0.9%
Infection	3	0.9%
Dehydration	2	0.6%
Constipation	1	0.3%
Fasting	1	0.3%
Gastro-oesophageal reflux disease (GORD)	1	0.3%
Hyponatraemia	1	0.3%
Multiple diagnoses	1	0.3%
Pseudo-obstruction	1	0.3%

## Discussion

In this retrospective descriptive cohort study, we conducted the largest investigation of CK screening for psychiatric inpatients to date. Crucially, we did not identify any cases of NMS, suggesting that, in our cohort, an admission CK lacks specificity as a screening investigation for this condition. Overall, in those with a raised CK, investigation of CK appeared to be mostly routine or untargeted and there was a low diagnostic yield. Our results call into question the utility of routine (untargeted) CK screening, given the uncertain patient benefit which results from abnormal results.

### Poor specificity of CK

CK is non-specific; it is either incidentally raised in many psychiatric inpatients [18], or raised for other reasons (including restraint or infection) [19–21]. The variety of diagnoses presented in Table 3 is in keeping with this; the only diagnoses occurring on more than one occasion were catatonia (3 cases; 0.9%), infection (3 cases; 0.9%) and dehydration (2 cases; 0.6%). Moreover, no single site of infection was present in more than 1 (0.3%) individual. CK rises may occur in more than one in ten patients with the commencement of antipsychotic medication (without features of NMS); [22, 23] it is suggested that this represents a separate, benign, syndrome with unclear cause, which is, however, distinct from NMS [22]. 

Although we did not identify any cases of NMS, catatonia was the equal-most frequent diagnosis (3 cases; 0.9%) made in the raised CK cohort. The exact relationship between catatonia and NMS has been a subject of debate for some time [7]. Catatonia is a psychomotor disorder with a diverse spectrum of clinical manifestations, including disturbances in speech, movement, volition, and autonomic changes [24, 25]. There is a considerable overlap in clinical features between the two syndromes, with some authors holding that NMS is a subtype of antipsychotic-induced catatonia [7, 24]. As such, it is unsurprising that the utility of CK as an investigation in catatonia has been examined, with mixed findings [24]. In the largest study of CK in catatonia, CK was significantly raised (2545IU/L) in those with catatonia (*n* = 74) compared with non-catatonic psychiatric patients (459IU/L) (*p* < 0.001), but with receiver operating characteristic analysis, the area under the curve (AUC) was 0.64 [25]. This suggests that CK would lack discriminatory ability in the diagnosis of catatonia.

Our results on the shortcomings of CK as a screening tool are consistent with those from other studies. Even in cases of suspected NMS, CK testing can have limited utility. A retrospective study [26] of 183 cases of suspected NMS showed poor accuracy for CK with cut-offs of both > 150U/L (41%) and > 1,000U/L (55%) for the detection of NMS, indicating poor discriminatory ability.

The utility of CK is further hindered in NMS, which has a very low prevalence (0.016% in inpatient settings) [5], and, hence, without any suggestive clinical signs or symptoms, the pre-test probability is low. This would translate to only a modest post-test probability in a test with a high specificity, but renders a test without a high specificity almost useless [15]. 

### Characteristics associated with raised CK

In our study, patients with incidentally raised CK were more likely to be male, and to have a psychotic disorder. The variation in CK activity between sexes has been previously characterized, with men on average having higher serum CK levels, which reflects their greater muscle mass [2]. 

The greater proportion of individuals with psychotic or affective disorders could perhaps reflect the greater potential for increased arousal and agitation in these disorders, with abnormally elevated CK activity previously demonstrated in a majority of inpatients with psychosis [12]. The exact mechanisms are unclear, but there is a consistent demonstration of elevated CK in inpatients with psychosis, with a suggestion that it could even function as a biomarker for illness severity [12, 27]. 

Antipsychotics have been demonstrated to independently increase CK, possibly by exerting effects on skeletal muscle cell membrane permeability [23]. CK levels have also been shown to be increased by restraint and intra-muscular injection, which may be more common in psychosis than other psychiatric conditions [28]. However, there could be a confounding effect present; the over-representation of psychosis in the cohort with raised CK could be due to the younger age of onset of psychosis and schizophrenia in men, which in turn increases the likelihood of greater average muscle mass [29, 30]. 

We report high prevalence of substance misuse (46.2%) in our raised CK cohort, with cannabis (63.9% of all substance misuse) the most common recreational drug. There is some evidence that cannabinoids specifically may increase CK levels in inpatients with psychosis, possibly due to their effects on skeletal muscle cells [31, 32]. 

### Suggestions for clinical practice

Psychiatrists may tend towards using CK as a confirmatory test for NMS, or even as a “rule out” test before commencing antipsychotics [19]. It may provide a falsely reassuring “objective’’ measure of the presence or absence of NMS. The results in this study suggest that a reconsideration of routine, undirected testing of CK (e.g., an “admission CK”) may be warranted.

Perhaps an apt comparison can be drawn with D-dimer. The D-dimer is marker of endogenous fibrinolysis, and is elevated in venous thromboembolism (VTE) [33, 34]. It has consistently demonstrated high sensitivity (> 95%) in detection of VTE and can be reliably used to exclude VTE in patients with a low pre-test probability [33]. However, the specificity of a D-dimer is only around 70%, making it unsuitable as a ‘rule in’ test for VTE [33]. Therefore, the utilization of D-dimer is incorporated into clinical probability rules for DVT and PE (e.g., Well’s score); [35, 36] if there is high clinical suspicion of VTE, a D-dimer does not need to be performed, as it lacks adequate negative predictive value (NPV) in these high-risk patients [33]. However, in those with low pre-test probability, the NPV would be sufficient to permit a D-dimer to be used to ‘rule out’ VTE [33]. In short, although the particulars of the test itself are unchanged, the application determines its utility.

Despite this, CK may still have clinical utility if deployed appropriately. NMS is a clinical diagnosis, and suspicion of its presence should lead to targeted investigations. Elevated CK remains the most common abnormal laboratory finding in NMS, and does, therefore, have a role in the investigation of suspected NMS [4]. Symptoms of NMS can be mild and other differential diagnoses can present in a similar fashion (e.g., central nervous system (CNS) infection or certain endocrine disorders) [4]. It is in these cases, where there is diagnostic uncertainty, that a directed CK test could have utility. It may not be able to function as a definitive investigation to include/exclude NMS, but it may help to guide diagnosis when used wisely. Likewise, judicious use of CK where there is existing clinical suspicion may have a role in assisting the diagnosis of catatonia [25]. 

### Limitations

This was a retrospective study of patient electronic records. Therefore, the data presented here were in turn reliant on the presence and accuracy of information recorded in clinical entries. It may be that interventions in response to a raised CK were occurring, but this was not being accurately reflected in the electronic clinical notes (e.g., frequency of observations was increased but was performed on paper charts).

CRIS database only contains electronic notes from one mental health trust (SLAM). If a patient had been transferred to a general hospital it was not possible to determine what interventions occurred. We had to rely on this information being repeated in the local electronic patient records by clinicians, which may not always have been completed or sufficiently robust.

South-East London has an incidence of psychosis considerably higher than other areas in the UK [37] South London is ethnically diverse, with 60.3% of people resident in the four London boroughs serviced by SLAM identifying as White [38]. The findings presented here may not therefore be generalizable to other areas of the UK or internationally.

## Conclusions

Overall, in this retrospective cohort study we have presented findings that highlight the limited clinical utility of routine, undirected testing of CK in patients admitted to a psychiatric inpatient unit. Based on these findings, we would recommend that mental health trusts consider revisiting any policies that suggest an admission CK should be routinely collected. We suggest that CK should be used in the presence of clinically-suspected pathology (e.g., NMS or catatonia) to help elucidate the diagnosis.

## Data Availability

Data are owned by a third party, Maudsley Biomedical Research Centre (BRC) Clinical Records Interactive Search (CRIS) tool, which provides access to anonymised data derived from SLaM electronic medical records. These data can only be accessed by permitted individuals from within a secure firewall (i.e. the data cannot be sent elsewhere), in the same manner as the authors. For more information please contact: cris.administrator@slam.nhs.uk.
